# Boosting the circularly polarized luminescence of small organic molecules *via* multi-dimensional morphology control†
†Electronic supplementary information (ESI) available. See DOI: 10.1039/c9sc01577a


**DOI:** 10.1039/c9sc01577a

**Published:** 2019-06-04

**Authors:** Kai Ma, Wenjie Chen, Tifeng Jiao, Xue Jin, Yutao Sang, Dong Yang, Jin Zhou, Minghua Liu, Pengfei Duan

**Affiliations:** a State Key Laboratory of Metastable Materials Science and Technology , Yanshan University , Qinhuangdao 066004 , P. R. China . Email: tfjiao@ysu.edu.cn; b CAS Center for Excellence in Nanoscience , CAS Key Laboratory of Nanosystem and Hierarchical Fabrication , National Center for Nanoscience and Technology (NCNST) , No. 11 ZhongGuanCun BeiYiTiao , Beijing 100190 , P. R. China . Email: duanpf@nanoctr.cn ; Email: liumh@iccas.ac.cn; c Beijing National Laboratory for Molecular Science , CAS Key Laboratory of Colloid Interface and Chemical Thermodynamics , Institute of Chemistry , Chinese Academy of Sciences , No. 2 ZhongGuanCun BeiYiJie , Beijing 100190 , P. R. China; d University of Chinese Academy of Sciences , Beijing 100049 , P. R. China

## Abstract

Achieving a higher dissymmetry factor is a crucial issue in developing circularly polarized luminescence (CPL) materials. Here, by tailoring the solvent composition and the morphology of the same chiral emissive small molecules (*R*- or *S*-SPAn), circularly polarized emission with a boosted dissymmetry factor (two orders) was realized. It was found that by regulating the water fraction in the mixed THF/H_2_O, we were able to achieve kinetic control over association of chiral emissive *R*- or *S*-SPAn into various nanostructures with 0D nanospheres, 2D nanoflakes and 3D stacked nanoflakes. These nanostructures are all CPL active. Remarkably, the dissymmetry factors of the nanostructures were significantly enhanced compared to those of the molecules and further boosted in different morphologies, from ∼10^–4^ (0D nanospheres) to 10^–3^ (2D flake) to ∼10^–2^ (3D nanoflakes). The enlarged *g*_lum_ value could be assigned to a good packing induced strong luminescence of an excimer. This strategy provides an efficient way to fabricate higher dissymmetry factor CPL organic nanomaterials by only changing the supramolecular architectures while using the same chiral small molecules.

## Abstract 2

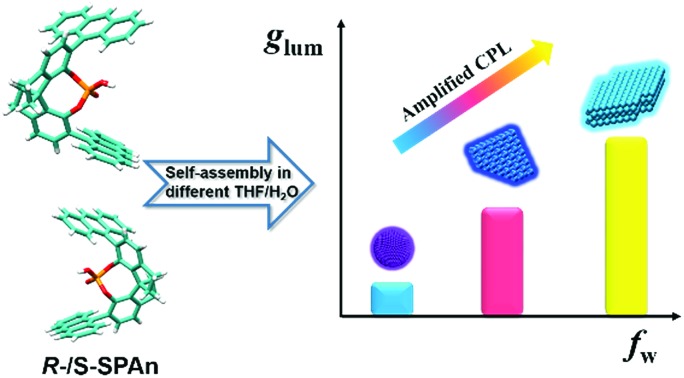
By regulating the composition of solvents, the assembled nanostructures of chiral molecules transformed from 0D nanospheres to 3D nanoflakes, which showed significantly amplified circularly polarized luminescence.

## Introduction

Chirality is a basic characteristic of nature which can be incisively and vividly exemplified by molecular, macromolecular or supramolecular levels of bioorganic systems, such as amino acids, enzymes, proteins, sugars, RNA and DNA.^1^–^8^ In recent years, excited state chirality, namely circularly polarized luminescence (CPL), has aroused much attention due to its potential for application in many fields.^9^–^20^ CPL originates from giving priority to one handed circularly polarized emission and the level of polarization is usually characterized by the luminescence dissymmetry factor (*g*_lum_). The value of *g*_lum_ ranges from –2 to +2 and the maximum value of |*g*_lum_| represents completely left or right-handed circularly polarized light.^21^–^23^ To broaden the application of CPL-active materials, it is necessary to pursue large *g*_lum_ of chiral luminescent systems. To date, the largest *g*_lum_ value with about 1.3 was obtained in lanthanide complexes, that is cesium tetrakis(3-heptafluoro-butylryl-(+)-camphorato) Eu(iii) complexes.^24^ On the other hand, in contrast to lanthanide complexes, research on simple organic molecules with CPL activity has become popular due to the inherent advantages of organic luminescent molecules.^25^,^26^ Compared with lanthanide complexes, the emission of pure organic CPL-active materials could be tuned more flexibly by regulating the electronic levels of the excited state. For instance, many strategies have been realized to modulate the electronic state of organic molecules, such as by chemical transformations of substituents, self-assembly and by changing the environment through external stimuli, *e.g.* temperature, pH, concentration, light, magnetism and solvents.^27^–^38^ Unfortunately, the *g*_lum_ value of organic systems is relatively low and generally falls in the range of 10^–5^ to 10^–3^, which precludes them from being the best candidates for CPL research. To amplify the luminescence dissymmetry factors, different approaches have been adopted, which include the formation of receptor–ion complexes, configurational changes upon binding with guest ions or molecules and self-assembly of chiral molecules.^39^–^46^ For example, Tang and co-workers, by introducing the concept of aggregation-induced emission, realized the amplification of *g*_lum_, which could be regarded as an excellent approach for fabricating efficient CPL-active organic solid materials.^25^,^47^,^48^ Very recently, we have demonstrated that energy transfer, including Förster resonance energy transfer and the photon upconversion process, could remarkably amplify the *g*_lum_ value.^49^–^51^ Kawai and co-workers have found that self-assembly could be used as an approach for amplifying the *g*_lum_ value.^52^ In addition, they have demonstrated that one-dimensional self-assembled nanostructures exhibited larger *g*_lum_ values than zero-dimensional aggregates. However, detailed demonstrations of morphology-dependent circularly polarized emission with regulated large *g*_lum_ values in pure organic molecule-based systems have rarely been reported.

**Scheme 1:**
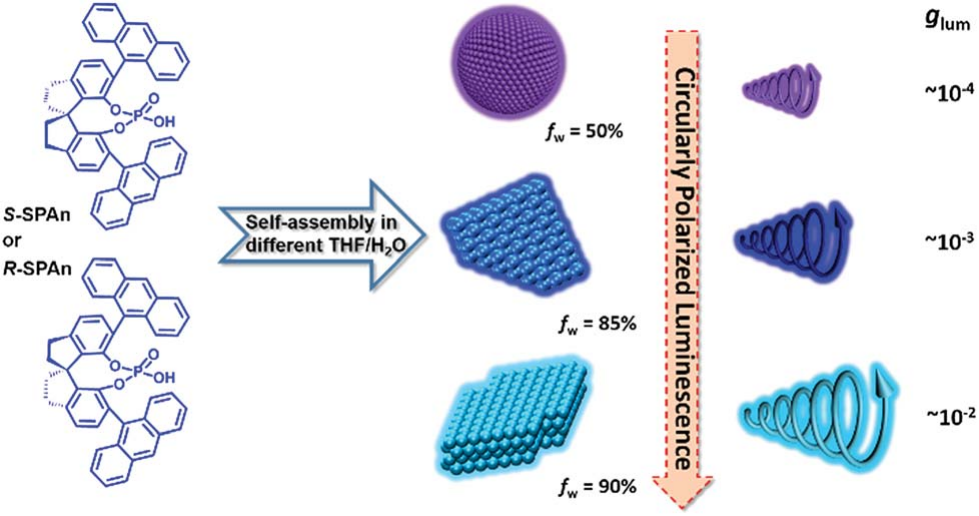
Self-assembly of chiral molecules into different nanostructures in mixed THF/H_2_O with different volume ratios. Upon increasing the volume fraction of water, assembled structures of nanoparticles, and 2D and 3D nanoflakes were obtained. The CPL of the aggregates obtained in various water fractions was gradually amplified and the *g*_lum_ value of 3D nanoflakes finally increased by two orders of magnitude in comparison with the monomeric molecules.

Herein, we report an interesting CPL-active organic system, which shows morphologically dependent CPL activity with controllable *g*_lum_ values, as shown in Scheme 1. This category of molecules was originally synthesized as a privilege catalyst for asymmetric reactions.^53^,^54^ Different morphological features of the nanostructures from the same chiral small molecule could be flexibly tuned by changing the mixing ratio of THF and water. Upon increasing the volume fraction of water, the nanostructures changed from 0D nanospheres to 2D and 3D nanoflakes. By trapping the chiral molecule association into different supramolecular architectures, the *g*_lum_ value of the specific nanostructures showed a remarkable amplification from 10^–4^ to 10^–2^. Particularly, compared with the monomeric state of the chiral emissive molecule, the *g*_lum_ value showed an amplification factor of two orders of magnitude. Thus, avoiding tedious organic synthesis, by simply regulating the composition of solvents, there still is great potential for enhancing the CPL activity.

## Results and discussion

**Fig. 1:**
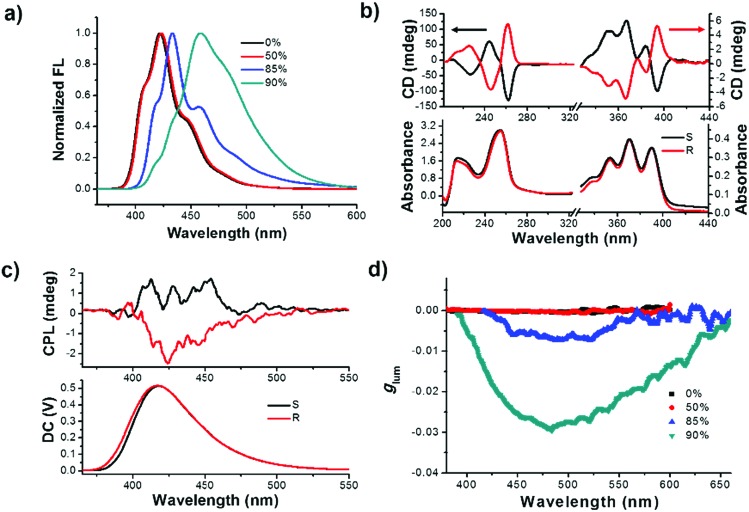
(a) Fluorescence spectra of *R*-SPAn aggregates in various *f*_w_ (*λ*_ex_ = 320 nm; intensity normalized at a maximum value). (b) CD spectra of *R*- and *S*-SPAn in pure THF. (c) CPL spectra of *R*-/*S*-SPAn in pure THF. (d) Dissymmetry factor *g*_lum_ of *R*-SPAn aggregates in various *f*_w_. Upon increasing the volume fraction of water, the maximum *g*_lum_ for *f*_w_ 90% was amplified two orders of magnitude in comparison with the *g*_lum_ for *f*_w_ 0% and 50%. ([*R*-SPAn] = 1.5 mM, *λ*_ex_ = 320 nm).

Initially, the primary photophysical investigations of monomeric and aggregated *R*- or *S*-SPAn were carried out by employing a series of optical testing methods. As shown in Fig. S1a,† upon increasing the volume fraction of water, the emission colour varied from deep blue to cyan under a UV lamp. The emission spectra also showed an obvious red shift. The maximum emission peak of *R*-SPAn in pure THF solution was located at 422 nm, which was identical to the one in the aggregate state in the mixed solvent (THF/H_2_O = 1/1) (Fig. 1a). Upon increasing the volume fraction of water (*f*_w_) to 85%, the emission spectrum showed a bathochromic shift to 432 nm with the emergence of a shoulder peak at 460 nm. When the water fraction reached 90%, the vibronic peaks tended to disappear and the maximum emission peak was located at 460 nm. Interestingly, the emission quantum yield (*Φ*_em_) showed an increasing trend from 0.44 (pure THF solution) to 0.6 (*f*_w_ 50%). However, after increasing the water fraction to 85%, the emission dramatically quenched (*Φ*_em_ = 0.04), which should be due to the aggregation-caused quenching of luminescence. Finally, upon increasing the water fraction to 90%, the quantum yield reached 0.13 (Fig. S1b and S2a†). As shown in Fig. S2b,† upon addition of water, the absorption spectra of *R*-SPAn showed a slight bathochromic shift and broadening, which indicated the formation of aggregates. The nature of different emitters of aggregates was investigated by emission lifetime measurement (Table S1†). The emission lifetime for *f*_w_ 0% and 50% exhibited a monoexponential decay with a lifetime of 8 ns for emission monitored at 422 nm, which could be attributed to the luminescence of monomeric molecules. Upon increasing the water fraction to 85%, the aggregates exhibited a fast biexponential decay and triple-exponential decay lifetime monitored at 432 nm and 460 nm, respectively. The emergence of new emissive species with a lifetime of about 2.3 ns and a longer lifetime of about 6.8 ns may be attributed to the luminescence of the aggregates and excimer, respectively. The lifetime for *f*_w_ 90% monitored at 460 nm exhibited a triple-exponential decay and the emergence of a long-lived lifetime of about 10 ns could further confirm the formation of an excimer.^55^–^57^ It could be concluded that the formation of the aggregates and excimer was responsible for the characteristic spectral features observed at higher water fractions.^58^

To test the chirality of the monomeric and self-assembled states, circular dichroism (CD) and CPL measurements were carried out under different conditions. In pure THF solution, the CD spectra of the monomeric molecule showed an obvious Cotton effect corresponding to the absorption bands (Fig. 1b). The CD signals from 200 to 300 nm could be assigned to the electronic transitions of the spiral chromophore with axial chirality,^59^ while the signals in the range of 350–400 nm were assigned to the anthracene units, respectively. Mirror-image CD spectra were observed for the *R* and *S*-enantiomers. Since the anthracene chromophores were achiral, the CD signals located at 350 nm to 400 nm could be assigned to the intramolecular chirality transfer from spiral chromophore to anthracene. The CD spectra of *R*- or *S*-SPAn under various conditions are shown in Fig. S3.† The CD intensity corresponding to the anthracene chromophore exhibited amplification accompanying the increasing water fraction. We used the absorption dissymmetry factor (*g*_CD_) to evaluate the change of chirality. The absorption of an asymmetric factor (*g*_CD_) is defined as *g*_CD_ = Δ*ε*/*ε* = 2(*ε*_L_ – *ε*_R_)/(*ε*_L_ + *ε*_R_), where *ε*_L_ and *ε*_R_ denote molar absorption coefficients of left and right circularly polarized light, respectively. The *g*_CD_ of the anthracene was amplified from 1.5 × 10^–4^ (390 nm) to 3.1 × 10^–3^ (412 nm) (Fig. S4†). These results indicated that the stronger exciton coupling in well-ordered structures could significantly amplify the *g*_CD_ by controlling the solvents. Thus, by controlling the morphology of nanostructures aggregated at various water fractions, amplified supramolecular chirality was obtained. The amplified *g*_CD_ laid the foundation for enhanced CPL as discussed below. The CPL spectra could provide more direct evidence for chiral dissymmetry in the fluorescence of the monomer and aggregates (Fig. 1c and S5†). Upon increasing the volume fraction of water, the intensity of the CPL was obviously enhanced. The extent of chiral dissymmetry in fluorescence is quantified using the anisotropy factor, *g*_lum_, of CPL, which is given by the equation *g*_lum_ = 2(*I*_L_ – *I*_R_)/(*I*_L_ + *I*_R_), where *I*_L_ and *I*_R_ are the intensities of the left- and right-handed circularly polarized emissions, respectively.^60^,^61^ As shown in Fig. 1d, in pure THF solution and *f*_w_ 50%, the *g*_lum_ values were about 2.1 × 10^–4^ and 2.4 × 10^–4^, respectively. Upon increasing the volume fraction of water, the *g*_lum_ value increased by an order of magnitude. The maximum *g*_lum_ values for *f*_w_ 85% and 90% were 7.2 × 10^–3^ and 2.9 × 10^–2^, respectively. The obtained *g*_lum_ value for *f*_w_ 90% in the pure organic systems was a relatively high value. The *g*_lum_ values of *S*-SPAn aggregated at various water fractions are shown in Fig. S6.† The order of magnitude of *g*_lum_ values of *S*-SPAn nanostructures at different water fractions was the same as that of *R*-SPAn. The obvious amplified *g*_lum_ values may have resulted from the strong intermolecular coupling in the nanostructures.

**Fig. 2:**
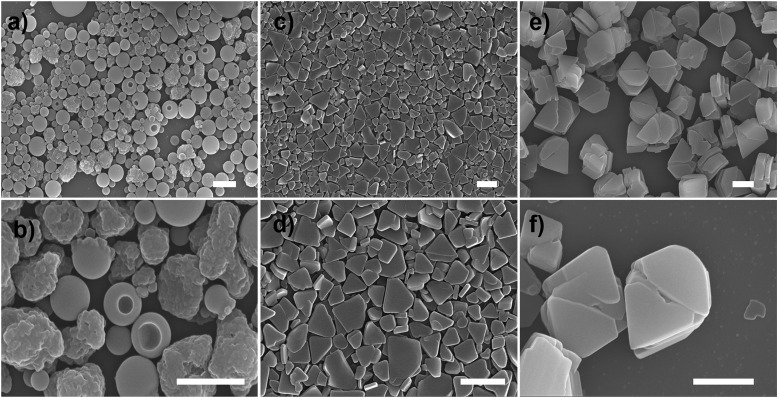
SEM images of nanostructures of *R*-SPAn in different *f*_w_: (a and b) 50%, (c and d) 85% and (e and f) 90%, respectively. ([*R*-SPAn] = 1.5 mM, scale bar 1 μm). Upon increasing the fraction of water, the morphologies transformed from 0D nanospheres with a rough surface or with a hole to 2D and finally to 3D layered nanoflakes.

Scanning electron microscopy (SEM) measurement was employed to investigate the morphological transformation of aggregates under different conditions. As shown in Fig. S7a,† the *R*-SPAn molecule formed amorphous structures upon evaporation of THF. When the water fraction reached 50%, two kinds of nanoparticles with a rough surface and with a hole could be observed (Fig. 2a and b). Upon increasing the fraction of water to 85%, two-dimensional nanoflakes were observed (Fig. 2c and d). Finally, when the water fraction reached 90%, three-dimensionally stacked flakes, which exhibit the largest *g*_lum_ value (Fig. 2e and f), could be obtained. The morphological transformation of *S*-SPAn nanostructures at various water fractions is shown in Fig. S6.† Thus, by varying the composition of the solvent, a dramatically changed CPL dependent on the morphological transformation could be obtained. We also tried higher volume fractions of water, such as 96% and 98%. Unlike the sample with a water fraction of 90%, the one with *f*_w_ 96% exhibited the structure of nanobelts, while hollow microspheres were observed in *f*_w_ 98% (Fig. S7b and c†). However, it is hard to get a plausible CPL signal under these conditions. This was due to the weak luminescence at a higher volume fraction of water.^62^

**Fig. 3:**
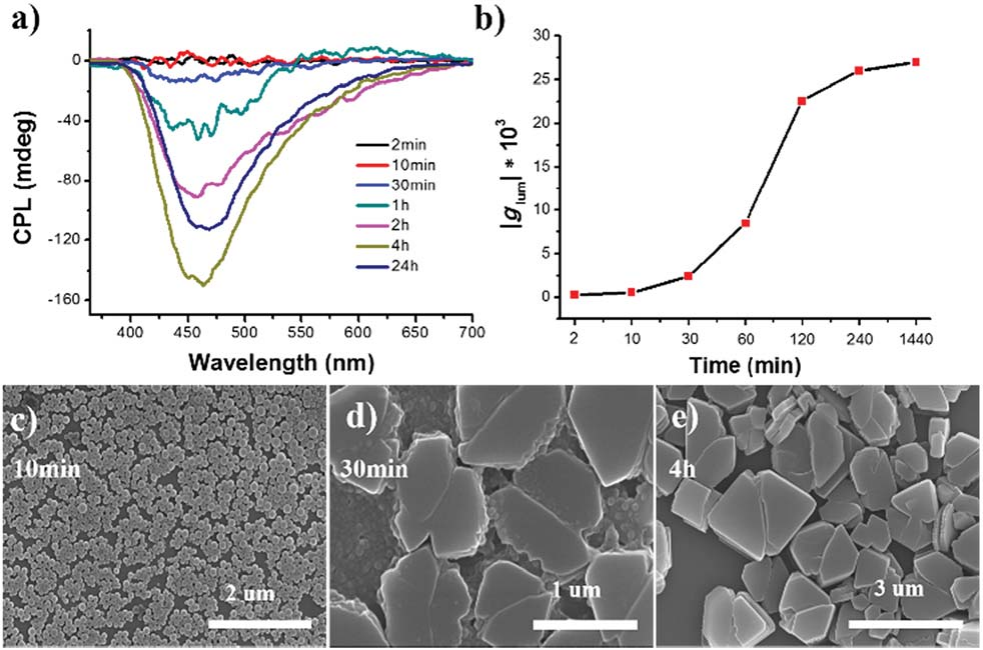
(a) Aging time-dependent CPL spectra of *R*-SPAn nanostructures in the water fraction of 90%. (b) The plot of *g*_lum_ value of nanostructures recorded at different times. Time-dependent SEM images of *R*-SPAn nanostructures in the water fraction of 90% at (c) 10 min, (d) 30 min and (e) 4 h, respectively. ([*R*-SPAn] = 1.5 mM, *λ*_ex_ = 320 nm).

The ripening process of *R*-SPAn in the water fraction of 90% was monitored by CPL measurement, as shown in Fig. 3a. The time-dependent CPL investigations in the water fraction of 90% showed that the CPL intensity dramatically increased. The time-dependent emission was also investigated, and it showed a similar tendency to CPL, as shown in Fig. S8.†

The *g*_lum_ value at different ripening times is shown in Fig. 3b. In the first two minutes, the *g*_lum_ value of the obtained sample was 2.2 × 10^–4^. After 4 hour ripening, the *g*_lum_ value reached 2.6 × 10^–2^, and remained at an almost constant value, confirmed by testing the sample after 24 hours (2.7 × 10^–2^). SEM was carried out to carefully investigate the morphological transformation at different ripening times. In the first 10 minutes, mono-disperse nanoparticles with a size distribution of about 140 nm were obtained, as shown in Fig. 3c. When the ripening time reached 30 minutes, the nanoparticles stacked together and fused to form a laminated flake structure with rough edges (Fig. 3d). After 4 hours, stacked 3D nanoflakes with a smooth surface were observed (Fig. 3e). Accompanying the morphology transformation from 0D nanospheres to 3D nanoflakes, the CPL activity also showed amplification. The *g*_lum_ value was amplified by two orders of magnitude to 0.027. The enhanced CPL activity of time-dependent morphology transformation in *f*_w_ 90% suggested that the CPL activity exhibited morphological dependence.

**Fig. 4:**
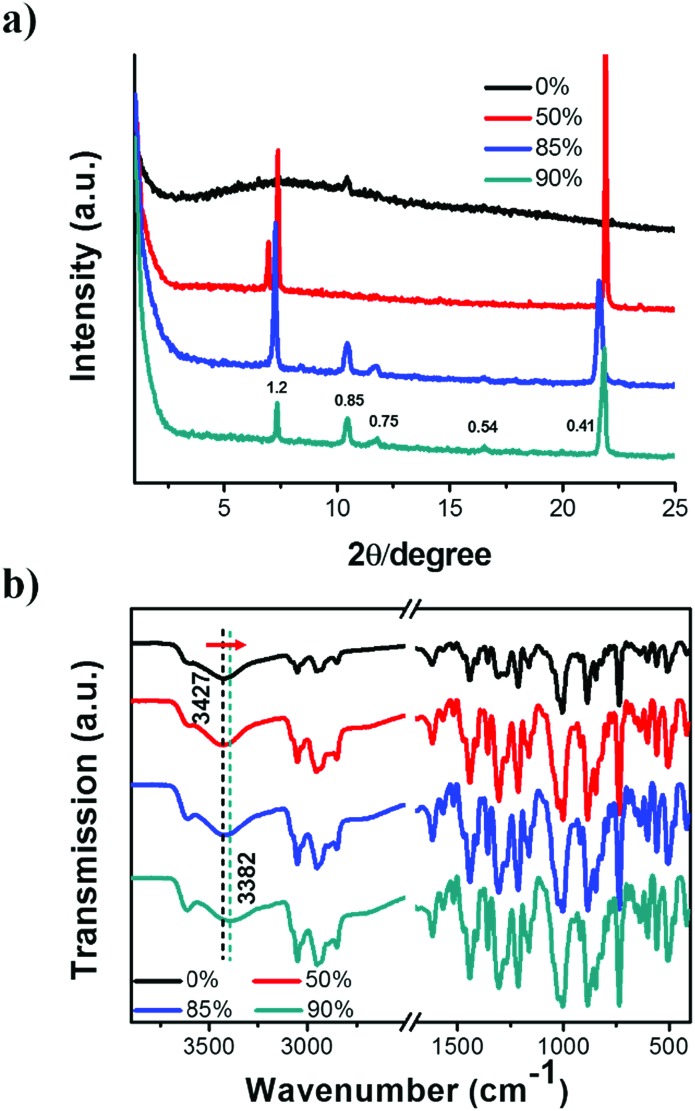
(a) XRD patterns and (b) FT-IR spectra of self-assembled *R*-SPAn nanostructures formed in various mixed solvents. The drop-cast film of *R*-SPAn solution (THF) was used as 0% for these tests. [*R*-SPAn] = 1.5 mM. The diffraction peaks of the water fractions of 85% and 90% showed a body-centred cubic packing of the *R*-SPAn molecules. Upon increasing the water fraction from 0% to 90%, the FT-IR peaks ascribed to the stretching vibration of hydroxyl obviously shifted toward lower wavenumbers.

To further clarify the formation of nanostructures with gradually enhanced CPL, X-ray diffraction (XRD) was carried out, as shown in Fig. 4a. The diffraction pattern of the *R*-SPAn cast film exhibited only one broad peak which indicated the formation of amorphous structures. For the *f*_w_ 50% sample, three diffraction peaks were observed at 2*θ* values of 7.01°, 7.37° and 21.94° with *d* spacings of 1.26 nm, 1.19 nm, and 0.41 nm, respectively. Clearly, upon increasing the volume fraction of water to 85% and 90%, three new diffraction peaks were observed at 2*θ* values of 10.46°, 11.71° and 16.55°. The whole diffraction peaks of *f*_w_ 90% gave *d* spacings of 1.20, 0.85, 0.75 and 0.54 nm with a *d* spacing ratio of about 
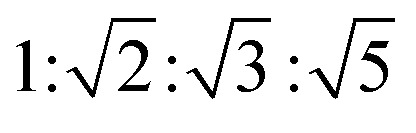
, indicating a body-centred cubic packing of the molecules.^63^ The first order diffraction peak of 3D nanoflakes was located at a 2*θ* value of 7.34° while the peak of 2D nanoflakes was located at a 2*θ* value of 7.24°, which indicated a closer molecular packing of the 3D nanoflakes than the 2D ones. In addition, the selected area electron diffraction (SAED) of the nanostructures obtained at *f*_w_ 85% and 90% showed ordered diffraction patterns (Fig. S9 and Table S2†). The obtained interlattice spacing was about 0.53–0.54 nm, which could be estimated from the result of XRD. These results suggested that the obtained 2D and 3D nanoflakes had ordered molecular packing and a crystalline nature to some extent.

To gain further insight into the non-covalent interactions, Fourier transform infrared (FT-IR) spectra of the nanostructures were obtained and are shown in Fig. 4b. The peaks located at 885 and 846 cm^–1^ could be attributed to the P–O stretching vibration (aromatic ring). The spectra with sharp peaks at around 1214 cm^–1^ could be ascribed to the P=O stretching vibration. In addition, the peak appearing at 1442 cm^–1^ could be ascribed to the stretching vibration of –CH_2_. The band appearing at 3052 cm^–1^ could be ascribed to the stretching vibration of unsaturated C–H single bonds.^64^ Upon increasing the fraction of water, the stretching vibration of the hydroxyl group of the phosphate moiety shifted toward lower wavenumbers, from 3427 cm^–1^ (*f*_w_ 0%) to 3382 cm^–1^ (*f*_w_ 90%). This was indicative of the existence of hydrogen bonding between intermolecular hydroxyl groups. Simultaneously, the peak became broader, which suggested the existence of an enhanced hydrogen bonding. These results indicated that the enhanced intermolecular hydrogen bond might be the main reason for enhancing the molecular stacking of 2D and 3D nanoflakes with relatively higher *g*_lum_ values.^65^

**Fig. 5:**
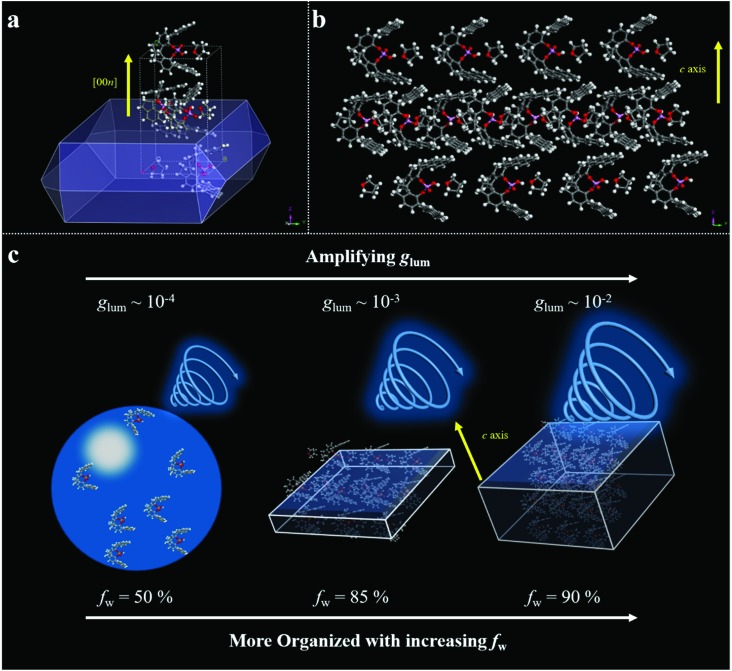
Molecular packing analysis results and speculated mechanism for the amplification of *g*_lum_. (a) Theoretically predicted growth morphology of an *S*-SPAn crystal based on the attachment energies calculated with Material Studio package. (b) Crystal packaging along the vertical growth direction. (c) Speculated mechanism for the amplification of *g*_lum_.

To gain a deep insight into the CPL amplification upon increasing the fraction of water, molecular packing analyses were carried out. We applied Materials Studio as a simulation tool for theoretically predicting the growth morphology of the *S*-SPAn single crystal. The simulation was performed using MS's morphology component based on the attachment energies. Firstly, the single-crystal of *S*-SPAn was incubated with THF/water mixed solvent. As shown in Fig. 5a, the crystal morphology of SPAn was plate-like, which was in agreement with the SEM results. The detailed crystallographic data are shown in Table S3.† Based on the XRD patterns, the growth in the thickness of the microplates was along the [001] direction, which could be attributed to the molecular packing based on π–π interaction of anthracene groups and hydrogen bonding between intermolecular hydroxyl groups as shown in Fig. 5b. With all of this in mind, we could speculate on a plausible mechanism for the amplification of CPL dissymmetry factor (*g*_lum_). When the water fraction was relatively low, the SPAn molecules aggregated in the form of microspheres, which was an amorphous packing mode and showed a relatively low *g*_lum_ (∼10^–4^). Upon increasing the water content, SPAn molecules could aggregate in the form of microplates in crystalline packing mode, and the organized packing could amplify the CPL dissymmetry factor (*g*_lum_ ∼ 10^–3^). Furthermore, when the water fraction was elevated to a higher level, more crystal units packed in the thickness direction, *i.e.*, more ordered structures, were involved in the CPL generation process. Thus, *g*_lum_ could be further amplified (*g*_lum_ ∼ 10^–2^).

## Conclusions

In summary, by tailoring the composition of the solvent (THF/H_2_O), the same chiral emitter *R*- or *S*-SPAn could be constructed into various nanostructures, including 0D nanospheres, 2D and 3D nanoflakes. Accompanying the morphological transformation, an amplified circularly polarized emission is observed. The maximum *g*_lum_ value could reach 0.029, which is a relatively large value in pure organic systems. The strong intermolecular interaction resulted in the formation of a more orderly and compact arrangement of nanostructures. The good packing of nanostructures induced a strong excimer emission, which could contribute a large *g*_lum_ value. The morphological dependence of emissive nanostructures on CPL activity can lead to controlled modulation of chiroptical properties, offering great potential for fabricating chiroptical organic nanomaterials.

## Experimental

### Materials

(11a*S*) and (11ab)-3,7-Di-9-anthracenyl-10,11,12,13-tetrahydro-5-hydroxy-5-oxide diindeno[7,1*de*:1′,7′-*fg*][1,3,2] dioxaphosphocin (*S*-SPAn and *R*-SPAn) were purchased from Daicel Chiral Compounds (Shang Hai) without further purification. Tetrahydrofuran was purchased from TCI. Milli-Q water (18.2 MO cm) was used in all cases.

### Preparation

*S*-SPAn and *R*-SPAn were dissolved in tetrahydrofuran (THF) with a volume of 1 mL, 500 μL, 150 μL and 100 μL, respectively. And then 500 μL, 850 μL and 900 μL of water were added to the latter three. Thus, the nanostructures with various morphologies could be obtained. The final concentration of *R*- or *S*-SPAn of all samples was 1.5 mM.

### Characterization

UV-vis spectra, fluorescence spectra and CD spectra were obtained using a Hitachi UV-3900, Zolix Omin-λ500i monochromator with a photomultiplier tube PMTH-R 928 and JASCO J-810 spectrometers, respectively. CPL measurements were performed with a JASCO CPL-200 spectrometer. XRD analysis was performed on a Rigaku D/Max-2500X-ray diffractometer (Japan) with CuKα radiation (*λ* = 1.5406 Å), operated at a voltage of 40 kV and a current of 200 mA. FTIR studies were performed with a JASCO FTIR-660 spectrometer. SEM was performed on a Hitachi S-4800FE-SEM with an accelerating voltage of 10 kV. TEM and selected area electron diffraction were performed on a transmission electron microscope, JEM1011. The fluorescence lifetime measurements were recorded on an Edinburg FLS-980 fluorescence spectrometer using time-correlated single photon counting.

## Conflicts of interest

There are no conflicts to declare.

## Supplementary Material

Supplementary information
