# Supplementary material for: Boosting the circularly polarized luminescence of small organic molecules via multi-dimensional morphology control
Source: Chem Sci. 2019 Jun 4;10(28):6821–7. doi: 10.1039/c9sc01577a (PMC6657416; doi:10.1039/c9sc01577a)
Supplement: Supplementary file 1 [file SC-010-C9SC01577A-s001.pdf]

Electronic Supplementary Material (ESI) for Chemical Science.
This journal is © The Royal Society of Chemistry 2019

Electronic supplementary information

Boosting Circularly Polarized Luminescence of Small Organic Molecules via Multi-dimensional Morphology Control

Kai Ma,[a,b] Wenjie Chen,[b,c,d] Tifeng Jiao,*[a] Xue Jin,[b] Yutao Sang,[b,d] Dong Yang,[b] Jin Zhou,[b] Minghua Liu,*[b,c,d] Pengfei Duan,*[b,d]

[a] State Key Laboratory of Metastable Materials Science and Technology, Yanshan University, Qinhuangdao 066004, P. R. China. E-mail: tfjiao@ysu.edu.cn
[b] CAS Center for Excellence in Nanoscience, CAS Key Laboratory of Nanosystem and Hierarchical Fabrication, National Center for Nanoscience and Technology (NCNST), No. 11 ZhongGuanCun BeiYiTiao, Beijing 100190, P. R. China. E-mail: duanpf@nanoctr.cn; liumh@iccas.ac.cn
[c] Beijing National Laboratory for Molecular Science, CAS Key Laboratory of Colloid Interface and Chemical Thermodynamics, Institute of Chemistry, Chinese Academy of Sciences, No. 2 ZhongGuanCun BeiYiJie, Beijing 100190, P. R. China.
[d] University of Chinese Academy of Sciences, Beijing 100049, P. R. China.

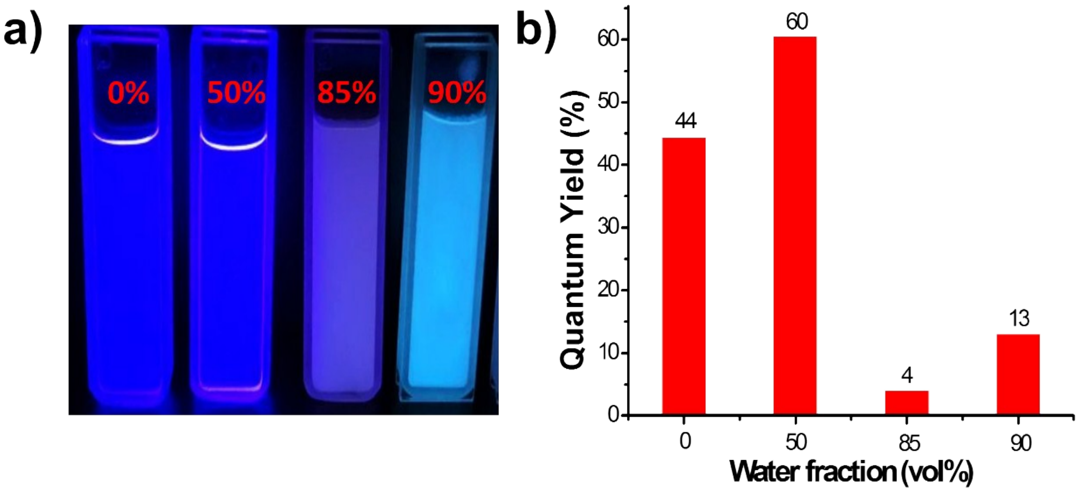

Fig. S1 (a) Photograph of *R*-SPAn prepared in various f_w under a UV lamp (λ_{ex}= 365 nm). (b) Quantum yield of *R*-SPAn prepared in different water fraction. With increasing the volume fraction of water, the emission quantum yield showed increasing and then dramatically quenched due to the aggregation-caused quenching of luminescence ([*R*-SPAn] = 1.5 mM, λ_{ex} = 320 nm).

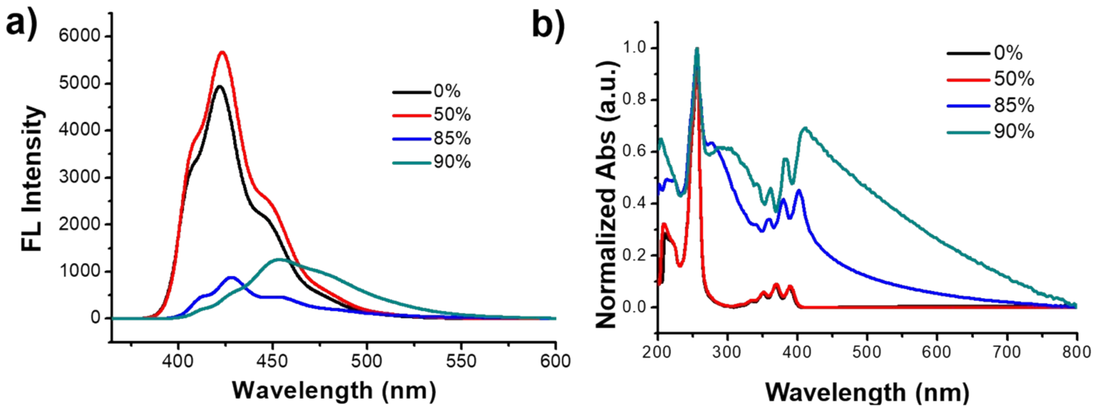

Fig. S2 (a) Fluorescence spectra and (b) UV-vis absorption spectra of *R*-SPAn prepared in different volume fraction of water ([*R*-SPAn] = 1.5 mM, λ_{ex} = 320 nm). The FL spectra showed obviously red shift and fluorescence quenching by increasing the fraction of water. The absorption spectra of *R*-SPAn showed a slight bathochromic and broadening, which indicated the formation of aggregates.

Table S1. The emission lifetime of *R*-SPAn prepared in various water fraction.

f_w	λ_{max} (nm)	τ (ns)	[(a)]τ_{avg} (ns)	CHICQ
0%	422	8		1.21
50%	422	8		1.23
85%	432	τ_1 0.85 (33%)	1.82	1
		τ_2 2.3 (67%)		
	460	τ_1 0.75 (18%)	2.8	0.99
		τ_2 2.3 (67%)		
		τ_3 6.8 (16%)		
90%	460	τ_1 0.96 (11%)	3.7	1.12
		τ_2 2.8 (70%)		
		τ_3 10.4 (19%)		

(a) Fluorescence lifetime (τ_{avg}) calculated using the equation τ_{avg} = A1τ1 + A2τ2 + A3τ3; λ_{ex} = 370 nm.

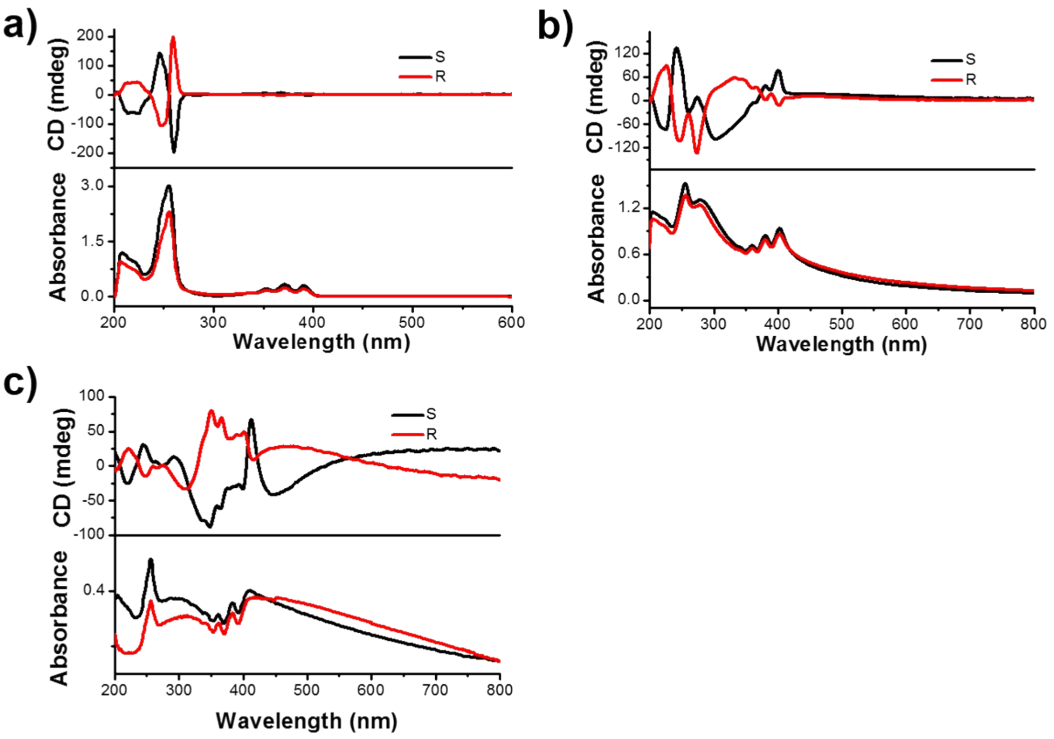

Fig. S3 CD spectra of nanostructures of *R*-/*S*-SPAn in various fraction of water (a) 50%, (b) 85% and (c) 90%. The CD peaks of all samples showed mirror-image signals ([*R*-/*S*-SPAn] = 1.5 mM).

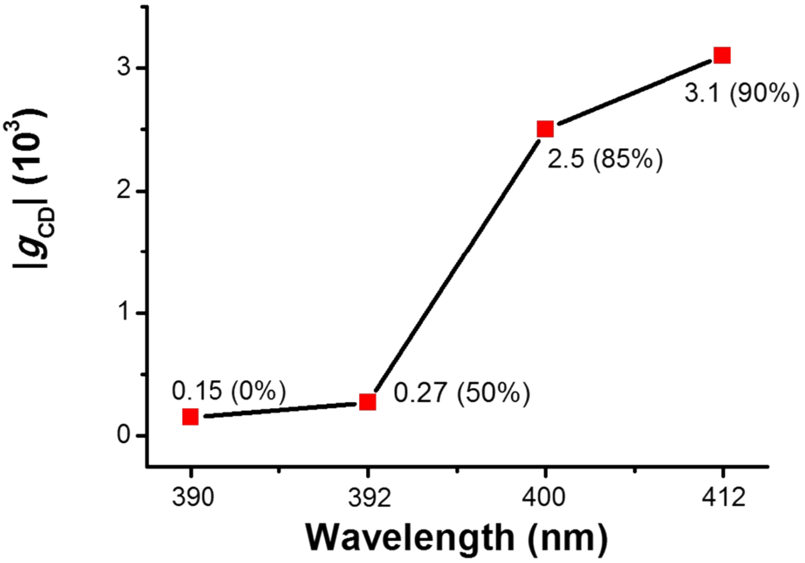

Fig. S4 The plot of g_{CD} of various nanostructures in different fraction of water. The g_{CD} value was recorded at one of the absorption peaks of the anthracene chromophore. With increasing the volume fraction of water, the g_{CD} showed obvious amplification. The g_{CD} of water fraction of 90% was 20 more times than the one of 0%.

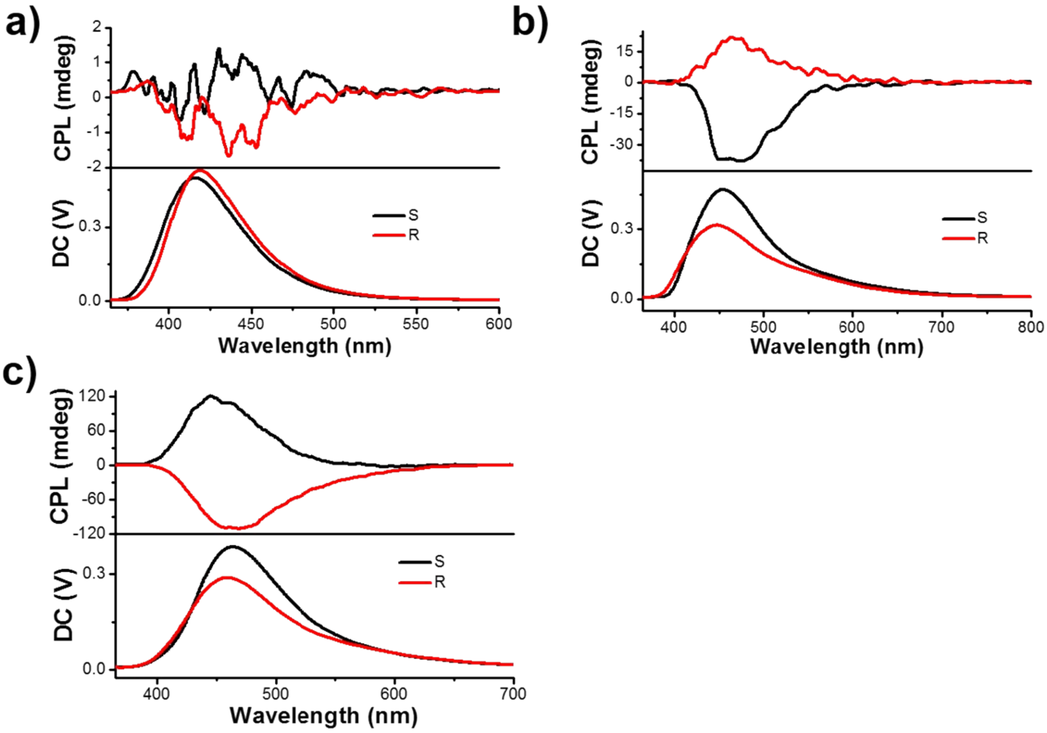

Fig. S5 CPL spectra of *R*- and *S*-SPAn nanostructures in various water fraction (a) 50%, (b) 85% and (c) 90% ([*R*-SPAn] = 1.5mM, λ_{ex} = 320nm). The CPL spectra showed mirror-image signals. With increasing the fraction of water, the CPL intensity was dramatically increased and the g_{lum} finally amplified two order of magnitude.

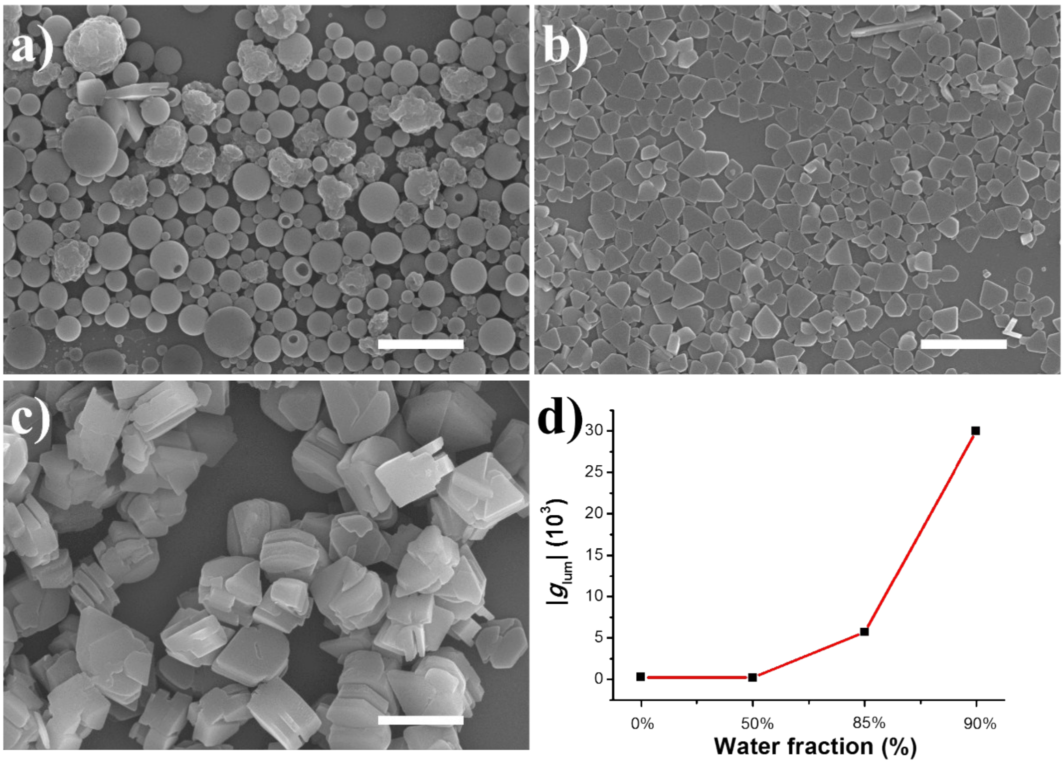

Fig. S6 SEM images of *S*-SPAn nanostructures in water fraction of (a) 50%, (b) 85% and (c) 90%. (d) Plot of g_{lum} value of *S*-SPAn nanostructures in various water fraction. The scale bar is 2 μm. The g_{lum} value of nanostructures finally amplified two order of magnitude.

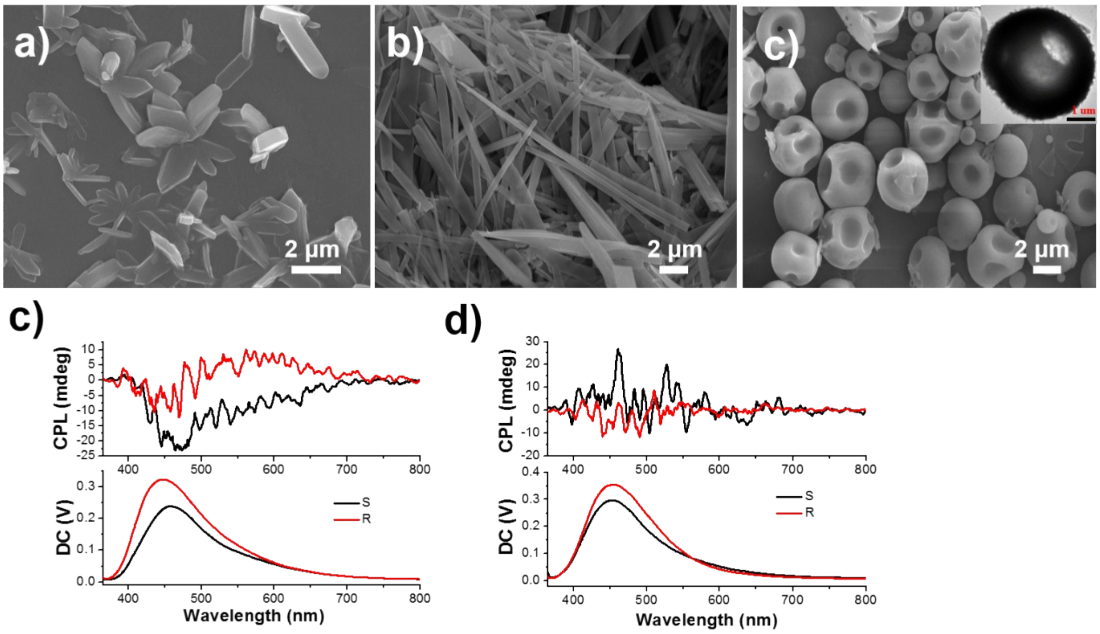

Fig. S7 SEM images of *R*-SPAn nanostructures in the water fraction of (a) 96% and (b) 98%. CPL spectra of *R*- and *S*-SPAn nanostructures at water fraction of (c) 96% and (d) 98% ([SPAn] = 1.5 mM, λ_{ex} = 320 nm). The 1D nanobelts and 0D spheres were obtained in the f_w 96% and 98%. The insert TEM image showed the spheres was hollow. However, it is hard to get a plausible CPL signal in f_w 96% and 98%

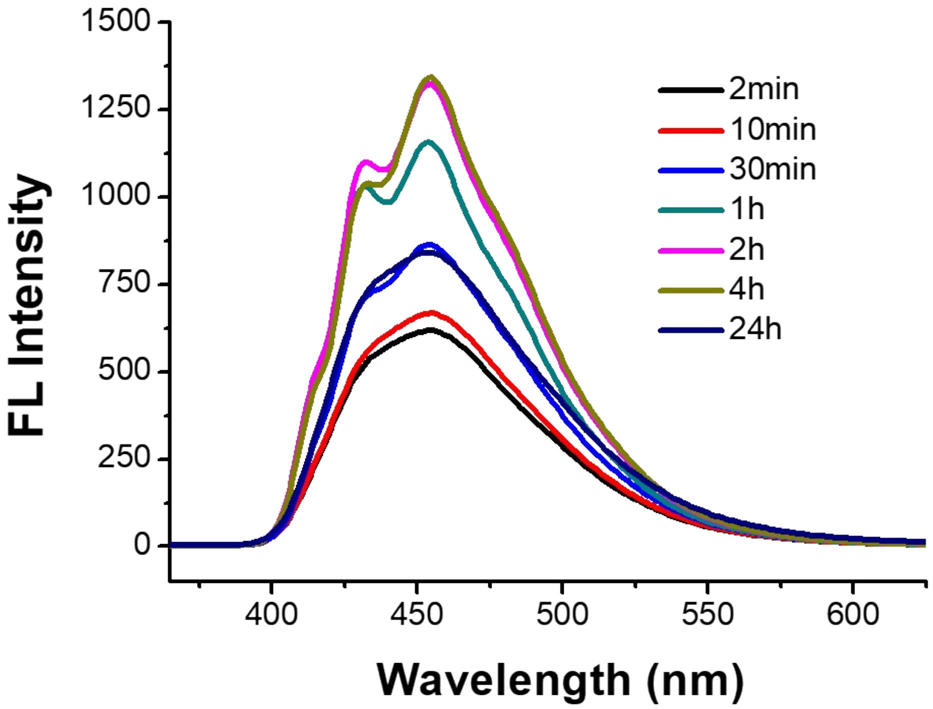

Fig. S8 Fluorescence spectra of *R*-SPAn nanostructures in the water fraction of 90% at different time ([*R*-SPAn] = 1.5mM, λ_{ex} = 320 nm). The FL intensity showed gradually increasing with prolonging the aging time.

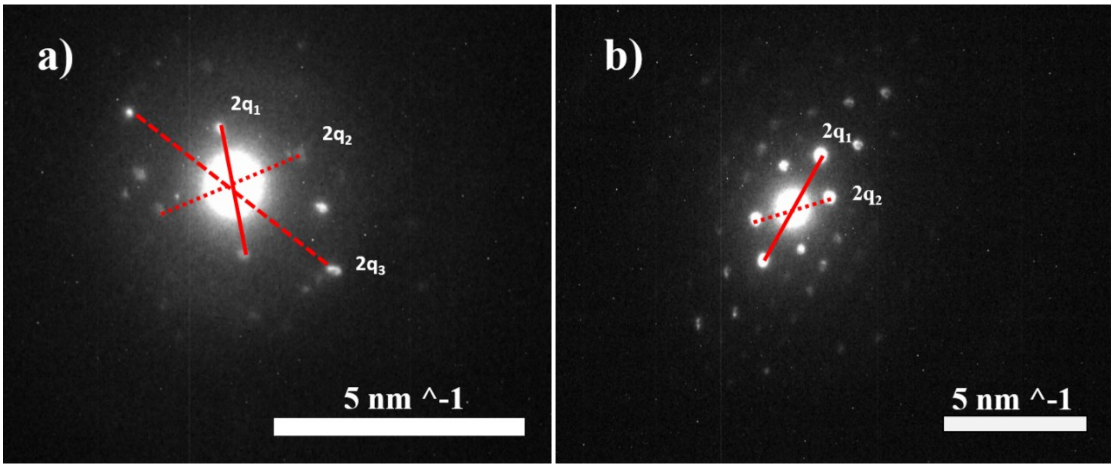

Fig. S9 Selective-area electron diffraction (SAED) of *R*-SPAn nanostructures in the water fraction of (a) 85% and (b) 90% ([SPAn] = 1.5 mM). The 2D and 3D nanoflakes showed ordered diffraction patterns.

Table S2. The *d*-spacing of SPAn nanostructures in various water fraction estimated from the SAED.

f_w %	d_1 (nm)	d_2 (nm)	d_3 (nm)
85 %	0.855	0.542	0.404
90 %	0.753	0.532	

Reciprocal vector $|\boldsymbol{q}| = 1/d$

Table S3. Crystallographic data for *S*-SPAn.

Sample	*S*-SPAn·THF
CCDC Number	1904614
Chemical formula	C98 H78 O10 P2
Formula weight	1477.54
Crystal system	Monoclinic
Space group	P 1 21 1
a (Å)	10.835(3)
b (Å)	14.503(4)
c (Å)	24.847(6)
α (°)	90
β (°)	100.702(3)
γ (°)	90
V (Å^3)	3836.5(16)
Z	2
D (g cm^{-3})	1.279
μ (mm^{-1})	0.121
T (K)	173.15
Goof	1.093
R1 (l>2σ(l))	0.0702
wR2(l>2σ(l))	0.1558
